# Non-Adherence with Physiotherapeutic Rehabilitation—A Cross-Cultural Adaption of Compliance Parameters into German

**DOI:** 10.3390/jpm13091353

**Published:** 2023-09-01

**Authors:** Hassan Tarek Hakam, Jonathan Lettner, Hannes Hofmann, Sebastian Kersten, Felix Muehlensiepen, Roland Becker, Robert Prill

**Affiliations:** 1 Center of Orthopedics and Trauma Surgery, University Clinic of Brandenburg, Hochstr. 29, 14770 Brandenburg an der Havel, Germany; hannes.hofmann@klinikum-brandenburg.de (H.H.); sebastian.kersten@mhb-fontane.de (S.K.);; 2 Medical School of Brandenburg, Fehbellinerstr 38, 16816 Neuruppin, Germany; jonathan.lettner@mhb-fontane.de (J.L.); felix.muehlensiepen@mhb-fontane.de (F.M.); 3 Center of Evidence Based practice Brandenburg (EBB), A JBI Affiliated Group, 14770 Brandenburg an der Havel, Germany; 4 Department of Orthopaedic Surgery, Sana Kliniken Sommerfeld, 16766 Kremmen, Germany; 5 Center for Health Services Research, Faculty for Health Sciences, University Clinic of Brandenburg, Seebad 82/83, 15562 Rüdersdorf bei Berlin, Germany

**Keywords:** non-compliance, non-adherence, physiotherapy, physical therapy, measurement, questionnaire, survey, translation, musculoskeletal

## Abstract

Background: Compliance with rehabilitative physiotherapeutic measures leads to an improvement in outcomes in patients suffering from a variety of musculoskeletal conditions. To date, a tool for assessing the parameters that lead to non-adherence to physical therapy does not exist in the German language. The objective of this paper is to cross-culturally adapt a non-compliance questionnaire to German. Methods: In reference to the “Guidelines for the Process of Cross-Cultural Adaption of Self-Reported Measures”, the questionnaire was translated into German followed by a back-translation into the original language. An expert committee met and refined the pre-final version. A preliminary version was handed out to patients for evaluation of the quality of the resulting German version. Results: After the forward- and back-translation of the questionnaire, some discrepancies were discovered between the translators on the one hand and between the back-translations and the original document on the other. The statistical analysis showed satisfactory results regarding the quality of the questionnaire. Conclusion: The translation and adaption of the items proved to have a high degree of reliability. The German version will be made available for German-speaking researchers and used for evaluating a mobile-application-based physical therapy regimen by the authors of the paper.

## 1. Introduction

Physical therapy is an important determinant influencing the outcome of many musculoskeletal diseases, including chronic low back pain [1] and early postoperative rehabilitation following total knee [2] or total hip replacement [3]. However, half of the affected patients are not active enough after total knee replacement [4]. This can negatively affect the overall outcome, with increased levels of pain, decreased functionality [2], reduced quality of life [5] and a rise in socioeconomic cost [6] observed in total knee replacement (TKR) patients. In order to determine the rationale behind a patient’s non-adherence, self-reported measures can be used. This appreciation of the patient’s perspective might help in tackling problems and challenges standing in the way of improving their health [7].

The research group at the Centre of Orthopaedics and Trauma Surgery is aiming to provide insights into patients’ noncompliance with rehabilitative measures after total knee replacement (TKR). A mobile-application-based physiotherapeutic program will be distributed to help enhance patient adherence. Telerehabilitation had previously been proven as an effective way to deliver treatment [8,9,10]. Although many papers have investigated the quality of early postoperative rehabilitative programs and exercise recommendations after TKR, relatively little work has been performed aiming to investigate the compliance of patients and its effect on the outcome.

As no validated German tools for the assessment of physiotherapy non-adherence exist, there was a need to adapt one from a foreign language. The aim of this study is to cross-culturally adapt the previously mentioned questionnaire to German. For that purpose, the questionnaire was translated to German according to the “Guidelines for the Process of Cross-Cultural Adaptation of Self-Report Measures”. This article validates the process already adapted by the American Association of Orthopaedic Surgeons (AAOS) [11].

## 2. Materials and Methods

**Figure 1:**
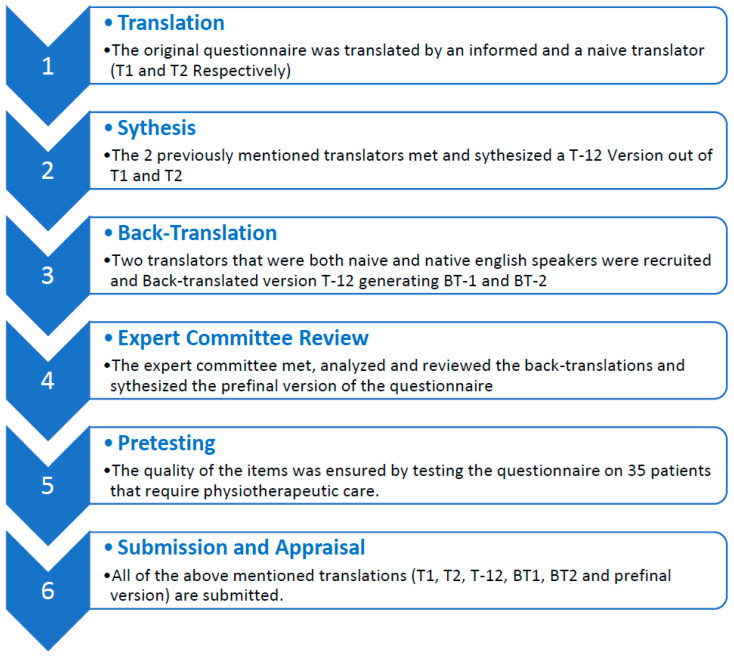
The stages of the cross-cultural adaption of the non-compliance questionnaire.

The “Correlates of Exercise Compliance in Physical Therapy” questionnaire contains four main domains: Discomfort, Barriers, Helplessness and Dependence. The domains are further broken down into three items. The items can be graded on a scale that ranges from one (agree) to four (do not agree). One represents the agreeableness of patients with a statement and four means that the patient disagrees with the statement. These elements were created in accordance with interviews made by clinicians and patients. The most prevalent answers were then included in the questionnaire. Steps included in the process are described below and summarized in Figure 1.

The original version is displayed in Appendix A.

### 2.1. Stage 1: Initial Translation

The survey items were to be translated according to the guidelines for cross-cultural adaption of self-reported measures [12]. For this purpose, two independent bilingual individuals that speak German as a first language were recruited to independently translate the original English version (source language) into a German version (target language). Comments regarding the nuances of individual elements were made during the process and alternative ways of comprehension were made by each translator. The first translator (version T1) was a physiotherapist working as a researcher at a university clinic. The translator was aware of the parameters examined by the questionnaire about the challenges of compliance. The second translator (version T2) (the naïve translator) was an elderly German woman with no clinical background. The choice was made based on her English comprehension ability, her fluency in German and the fact that she was representative of the target population intended to be examined by this questionnaire. No academic motives were perused by the translator working on T2.

The T1 and T2 documents are displayed in Appendix B.

### 2.2. Stage 2: Synthesis of the Translations

The two parties involved in the translation of T1 and T2 met to reach a consensus and synthesise a German version of the intended questionnaire. A third party observed the process and noted the differences and discrepancies between the two translators. The first common translation, T-12, was generated. Where little to no discrepancy between the translations were found, translators did not discuss the elements in depth. Those items were directly adopted into the survey.

### 2.3. Stage 3: Back-Translation

Two translators, who were blinded with respect to the original questionnaire as well as to the concepts tested by that questionnaire, were handed the synthesised translation T-12. Each “naïve” translator generated a back-translation of the survey. The two back-translators met, exchanged their ideas, discussed discrepancies and generated a new English version of the document. This step ensured that the agreed-upon version reflected the items of the original document, pointed out the flaws in the translation and the linguistic barriers that prevented the clear presentation of some items.

The first back-translation (BT1) and the second back-translation (BT2) are displayed in Appendix C.

### 2.4. Stage 4: Expert Committee

The expert committee was made up of a group of health care professionals as well as experts in qualitative studies. It included a bilingual attending surgeon with a medical degree from the United Kingdom, a bilingual German physiotherapy researcher with many publications in English, a bilingual orthopaedic surgery research fellow with a degree from an English-speaking University and a bilingual qualitative researcher. The naïve translators of T2, BT1 and BT2 were invited to attend the expert meeting and engage in the design of the final form. They will be mentioned in the acknowledgements section. The experts considered all previously mentioned versions of the translations and compared them with the original document. This step of the process concluded the pre-final version of the questionnaire.

### 2.5. Stage 5: Testing the Pre-Final Version

According to the “Guidelines for the Process of Cross-Cultural Adaption of Self-Report Measures” by Beaton et al. [12], the pre-final version should be tested on 30 to 40 subjects. The testing took place in paper form. The pre-final version was printed out and distributed to ward patients undergoing rehabilitative care. The patients were asked to grade each item of the survey and to highlight questions that presented comprehension difficulties. 

According to Beaton et al. (2000), the pretesting stage should not warrant any changes in the translation of self-reported measures [11]. Therefore, the pre-final version, generated by step four, will be taken as is. To assess the quality of this translation, questions that represented comprehension difficulties will be presented in this section and discussed later.

## 3. Results

### 3.1. Results of the Synthesis of the Translation (T-12)

Disagreements included points five, six, eight and eleven of the questionnaire survey. The T1 translator tried to convey the cultural equivalent of the phrases, while the T2 translator tried to communicate the literal translation of the items. After discussing the issues at hand, it was agreed to use the cultural equivalent of the items since the patients need to assess their own physical condition. Implicating the cultural rather than literal approach ensured that patients would comprehend what was being assessed.

### 3.2. Results of the Expert Committee Evaluation (Step 4)

The most outstanding discrepancy to the original version seemed to be the reference to the word exercise. In the German language, the most common literal translation of the word “Üben” is “practice” [13]. This does not refer to the physical nature of what is being practiced. However, in the absence of a German translation that clearly depicts the physical nature of the word, the fact that the second back-translation BT2 exclusively referred to the word “exercise” and that the document will clearly emphasise the bodily nature of what is being assessed, the phrasing was not altered. 

The second element of disagreement was the time frame of item four. While “having too little time” (in the original document) or “not enough time” (in BT1) refer to relative measures, “not having time” (in BT2) refers to an absolute measure. The item will have a grading of one to four and thereby indicates a measure that is variable and not absolute. Since “zu wenig” refers to “too little” and one back-translator (BT1) expressed that relativity, this translation will also be adopted in the final version.

The third point was the negative phrasing of item 9 in the back-translation. “I can do little” was back translated into “I cannot do much on my own” (BT1) and “I cannot do a lot on my own” (BT2), since “zu wenig” literally translates to “too little”. Since the grading is on a one to four scale with one meaning “agree” and four meaning “disagree”, to avoid confusing the patients with double negatives, the translation will be taken as is.

The fourth and last item to be discussed is the word “complaint”. The literal translation of the word complaint is “Beschwerde”. Since the two back-translators referred to a “problem” (BT1) or a “health problem” (BT2), the meaning was well-conveyed. Yet again, it was agreed that the literal translation of the word is to be used.

The pre-final version of the questionnaire is displayed in Table 1.

### 3.3. Results of the Pre-Final Version Testing

Thirty-one patients filled out the questionnaire and highlighted items where difficulties in comprehension arose. Based on the retrieved information, a statistical analysis was conducted to identify the questions that revealed comprehension problems. The comments from patients that were deemed most relevant are presented in Table 1. Other comments are presented and discussed in the discussion section.

Comments regarding item 7 and items 10 and 11 are further discussed in the comments section. All other comments about general aspects of the survey are displayed in Appendix D.

A simple statistical analysis was performed to evaluate the responses of previously mentioned patients in terms of motives to not comply with physical therapy, in accordance with the questionnaire. The results are presented in Table 2.

Sixteen male patients (52%) and fifteen female patients (48%) between the ages of fifty-six and eighty-seven years old (mean age of sixty-six) were surveyed. Eight patients had a total and four a partial knee replacement. Thirteen patients had undergone total hip replacement and one patient had a revision surgery after a total hip replacement. Seventeen patients stated that they received physical therapy in the past, twelve patients stated that they never had physiotherapy and three patients did not answer.

## 4. Discussion

The primary result of this study yielded a German version of the non-compliance questionnaire. This will enable clinicians to evaluate the reasons behind the non-adherence of patients to physiotherapeutic care in Germany and German-speaking countries. Using this tool will enable clinicians to develop strategies that aim at overcoming barriers of adherence to physical therapy.

The synthesis of a common forward-translation proved to be challenging. While the translator of T1 tried to convey a cultural translation of the items, the T2 translator tried to provide a literal translation. Putting the items in the context of a colloquially German-speaking community was deemed to have a higher priority [12].

The findings of testing Stage 5 revealed two main challenges. The first challenge came with the word to afford. Since the financial aspect was not clearly implied, one patient made a comment that the respective answer was based on the financial aspect of treatment. Another challenge was the discrimination between item 10 and item 11. Whilst item 10 suggests a total dependency on a physiotherapist, item 11 suggests that treatment directed by a physiotherapist is the most important aspect of rehabilitative care. Despite being doubtful about the three items, all patients correctly interpreted the meaning (as seen in the comment section), and given the fact that the translation methodology concludes the step with the synthesis of the expert committee, no further changes were undertaken.

The most common problems with adherence seemed to be the excessive reliance on the physiotherapist. This goes in hand with previous research conducted on the topic [13]. These results, however, are not representative and a conclusion cannot be made based on these findings. This is due to the small sample size as well as other factors, such as demographics (age range), geographical constraints (ward patients of the orthopaedics department) and diversity regarding the musculoskeletal conditions [14].

Finally, a clear relationship between compliance with physiotherapy and better outcomes has not yet been established. The literature only provides grounds for the idea that most patients are not active enough to maintain physical health [4]. Additionally, clinical studies do not objectively quantify compliance, as they rely on patient self-evaluation. Using this questionnaire, the authors of this paper will aim to identify the link between compliance, outcomes and mobile-application-based rehabilitation. 

Elements included in the resulting table of the study primarily based their findings on interviews with patients in ambulatory treatment in private practice in the Netherlands [7]. Most of the elements can be included in our survey, as they are representative of patient attitudes regardless of the setting in which treatment takes place. However, our research will be performed using data from patients at the ward of the Centre for Orthopaedics and Trauma Surgery at a university clinic. The patients will have received a TKR and be bedridden immediately after surgery. Therefore, item four, indicating the limited time of the patients, will be removed. In addition, item seven, referring to the affordability of the treatment, will be omitted.

After conducting a literature review, items including inpatient-specific factors will be discussed after a literature review by a panel of experts and included as an extension to the translated items. These items might include the patient–physician relationship [15], factors related to the environment in which physiotherapy takes place [16] and patient demographics, as well as familial support [4]. A more detailed review of the literature will be conducted. The resulting concepts will be brought together, arranged, prioritised and discussed by the group of specialists. The work of Ikart (2019) will be used to guide the expert review and pretesting of the synthesised questions [17].

In addition, aspects concerning the use of technology in guiding the rehabilitation of patients should be included to offer insight into the patients’ perspective. Since most patients with osteoarthritis are elderly, the usage of an app-based program might prove challenging [18]. Taking patient input into account will ensure the development of telerehabilitative methods that prove useful. Some improvements targeting the usability of mobile applications for the elderly were previously made [19]. Other studies reviewed existing applications for rehabilitation after total knee and hip replacement and made recommendations for future mobile application design [20]. The Centre of Orthopaedics and Traumatology at our institute is planning a randomised controlled trial using a mobile health application for rehabilitative purposes. The authors of this paper hope to add further recommendations that allow developers to optimise their physiotherapeutic apps designed for rehabilitative purposes after orthopaedic surgery.

Comments stated by patients were often out of focus with the questionnaire itself. These provided insight on the attitudes and beliefs patients hold towards physical therapy. An example would be a comment stating that “physiotherapists can only do as much as teach the patients about the required exercises” and “that the burden of completing the exercises depends on the patients themselves”.

### Limitations

The main limitation of this study was the contrast between the literal translation and the cultural translation. While some elements can be translated as is, yielding the same intended meaning, other elements proved more challenging. This resulted in adopted words having a slightly different literal interpretation. However, the context in which they were put in conveyed the meaning of the individual elements.

## 5. Conclusions

A cross-cultural adaption and validation of the survey mentioned in the study “Correlates of Compliance in Physical Therapy” was realised according to the above-mentioned guidelines. The resulting translation will help researchers identify problems faced by patients in rehabilitative care in German-speaking countries. This could lead to the development of strategies that increase the participant’s adherence with the recommended physiotherapeutic care, and thereby potentially ameliorate the outcome for a number of musculoskeletal conditions.

## Figures and Tables

**Table 1:** Details the final version (pretesting) translated elements and presents the number of participants who encountered comprehension problems, as well as their respective comments.

Item Number	Original English Version (Final German Version Provided below Each Element)	Percentage of Problems	Comments
1	I get tired from exercising	0	None
	Ich werde vom üben müde.		
2	The exercises are too difficult	0	None
	Die Übungen sind schwierig.		
3	The exercises are painful	0	None
	Die Übungen sind schmerzhaft.		
4	I have too little time	0	None
	Ich habe zu wenig Zeit um zu üben.		
5	Exercises do not fit daily routine	0	None
	Die Übungen passen nicht in die tägliche Routine.		
6	I often forget to exercise	0	None
	Ich vergesse oft zu üben.		
7	I cannot afford to exercise	1	Leisten?
	Ich kann es mir nicht leisten zu üben.		
8	Exercising will not help much	0	None
	Die Übungen werden nicht viel helfen		
9	I can do little by myself	0	None
	Ich kann wenig alleine machen.		
10	Recovery depends on the Physiotherapist	1	What is he differences to item 11?
	Die Genesung hängt vom Physiotherapeuten ab.		
11	The therapist is more important	1	Difference to item 10
	Der Physiotherapeut ist wichtiger		
12	My complaints will disappear without exercising	0	None
	Meine Beschwerden werden sich auch ohne Übungen lösen.		

**Table 2:** An analysis of the responses of patients to the non-compliance questionnaire expressed as a percentage per total number of patients.

Item Number	1 (Disagree) (%)	2 (%)	3 (%)	4 (Agree) (%)
1	58	26	9	3
2	19	35	26	3
3	19	23	35	16
4	84	10	3	0
5	71	10	10	6
6	58	23	13	6
7	77	10	3	10
8	45	13	10	19
9	39	26	10	23
10	29	19	10	32
11	19	13	16	38
12	41	10	13	16

In this statistical analysis, the percentage is expressed per total number of patients.

## Data Availability

Additional data are available upon request from the corresponding authors.

## Appendix A

Original table representing items in English. Please refer to the manuscript text and references for more details.

**Item Number**	**Text in the Original Language**
1	I get tired from exercising.
2	The exercises are too difficult.
3	The exercises are painful.
4	I have too little time.
5	Exercises do not fit in daily routine.
6	I often forget to exercise.
7	I cannot afford to exercise.
8	Exercising will not help much.
9	I can do little by myself.
10	Recovery depends on the Physiotherapist.
11	The therapist is more important.
12	My complaints will disappear without exercising.

## Appendix B

Table Representing the Forward Translation T1 and T2 into German.

**Translation T1**	**Translation T2**
1 Ich werde vom üben müde	1 Ich werde vom üben müde
2 Die Übungen sind schwierig	2 Die Übungen sind schwierig
3 Die Übungen sind schmerzhaft	3 Die Übungen sind schmerzhaft
4 Ich habe zu wenig Zeit	4 Ich habe zu wenig Zeit um zu Üben
5 Die Übungen passen nicht in meine Routine	5 Die Übungen passen nicht in die tägliche Routine
6 Ich vergesse es oft zu üben	6 Ich vergesse oft zu üben
7 Ich kann es mir nicht leisten zu üben	7 Ich kann es mir nicht leisten zu üben
8 Üben wird mir nicht helfen	8 Die Übungen werden nicht viel helfen
9 Ich kann wenig alleine machen	9 Ich kann wenig alleine machen
10 Die Genesung hängt vom Physiotherapeuten a	10 Die Genesung hängt vom Physiotherapeuten ab
11 Der Physiotherapeut ist am wichtigsten.	11 Der Physiotherapeut ist wichtiger
12 Meine Beschwerden werden sich auch ohne Übungen lösen	12 Meine Beschwerden werden sich auch vermindern ohne zu üben.

## Appendix C

Table Representing the Backward Translations BT1 and BT2.

**Back-Translation BT1**	**Back-Translation BT2**
1 I get tired from exercising	1 I get tired from practicing
2 The exercises are difficult	2 The exercises are difficult
3 The exercises are painful	3 The exercises are painful
4 I don’t have enough time (to exercise)	4 I don’t have enough time
5 The exercises don’t fit into my daily routine	5 The exercises don’t fit into my daily schedule
6 I often forget to do the exercises	6 I often forget to practise
7 I cannot afford to do the exercises	7 I cannot afford to practise
8 The exercises will not help much	8 The exercises will not help much
9 I cannot do much on my own	9 The recovery depends on my physiotherapist
10 Recovery depends on the physiotherapist	10 The recovery depends on my physiotherapist
11 The physiotherapist is more important (than the exercises)	11 The physiotherapist is more important
12 My health problems will resolve without exercise	12 The physiotherapist is more important

## Appendix D

### Comments Provided by Patients in the Corresponding Section of the Questionnaire

Patient 1: “The main factor influencing healing is the attitude of the patients themselves. A physiotherapist can only do as much as instructing us on how to do the exercises properly. Executing the exercises on a regular basis is our responsibility”.
Patient 2: “Simple yes or no questions would be better, although I found no difficulties in answering the items.”
Patient 3: “I do not consider the physiotherapist to be essential in the daily execution of exercises. However, exercising with someone, maybe like a group of people, would be much more motivating than doing so alone”.
Patient 4: “I think we need a paper-based program than details the correct way of performing an exercise since I am afraid to do it in a wrong manner and hurt myself”.
Patient 5: “I try to do my exercises routinely, but Pain presents rapidly and inhibits further repetitions.”
Patient 6: “I think these questions are subjective and answering them might depend on my mood. Yesterday I was feeling pain so I would have answered them differently”.
